# Supplementary material for: Variablity of Mechanical or Tissue Valve Implantation in Patients Undergoing Surgical Aortic Valve Replacement in Spain: National Retrospective Analysis from 2007 to 2018
Source: J Clin Med. 2021 Jul 21;10(15):3209. doi: 10.3390/jcm10153209 (PMC8347167; doi:10.3390/jcm10153209)

Supplemental Material

Supplemental Figure S1. Number of TAVR & SAVR per million inhabitants in Spain.

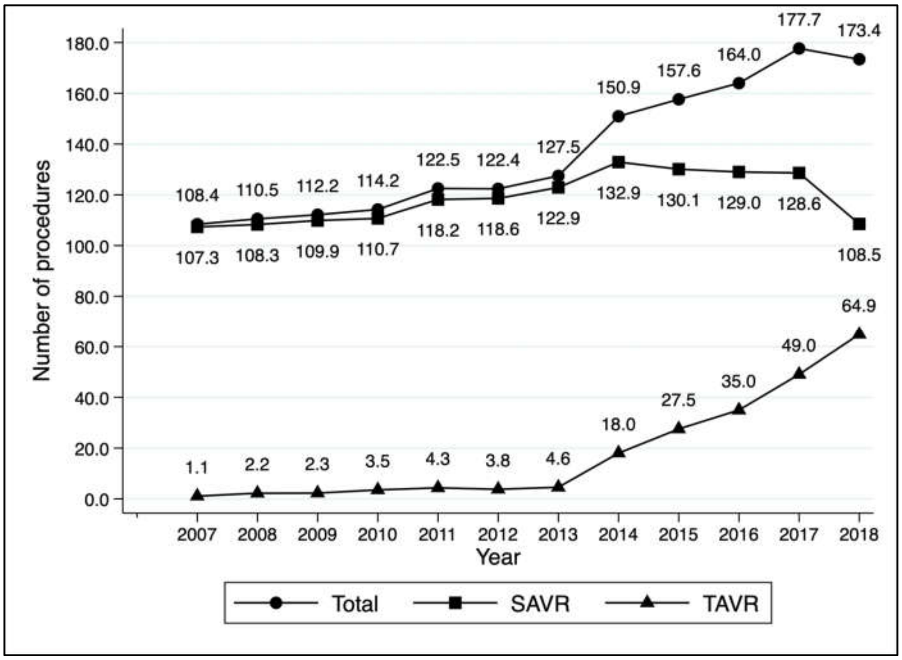

SAVR: Surgical aortic valve replacement. TAVR: transcatheter aortic valve replacement.

Supplemental Figure S2. Number of TAVR, Tissue Valves and Mechanical valves per million inhabitants in Spain.

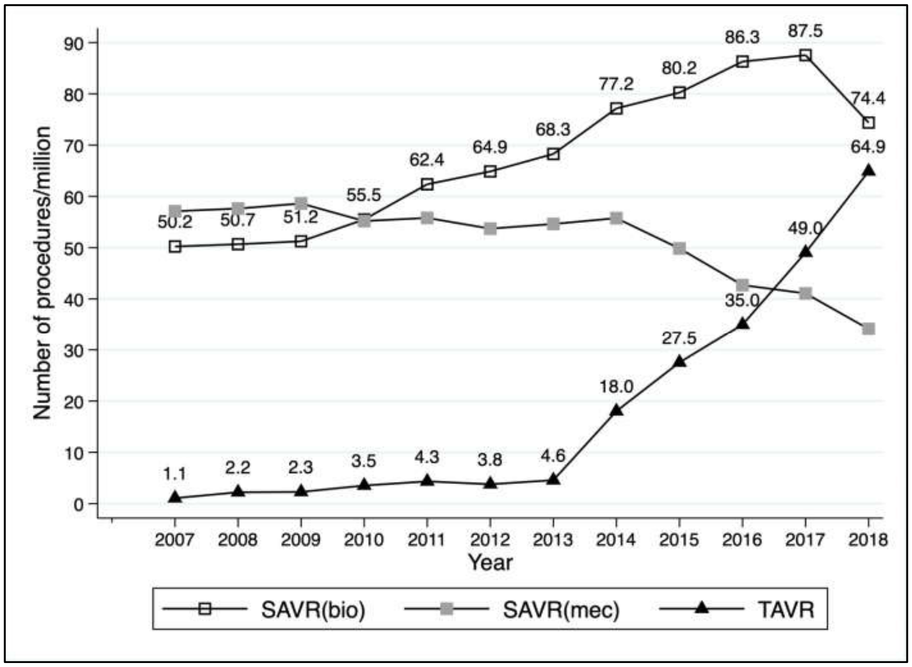

SAVR: Surgical aortic valve replacement. TAVR: transcatheter aortic valve replacement. Bio: tissue valve. Mec: mechanical prosthesis

Supplemental Figure S3. Mechanical or tissue valve replacement/ million inhabitants according to sex.

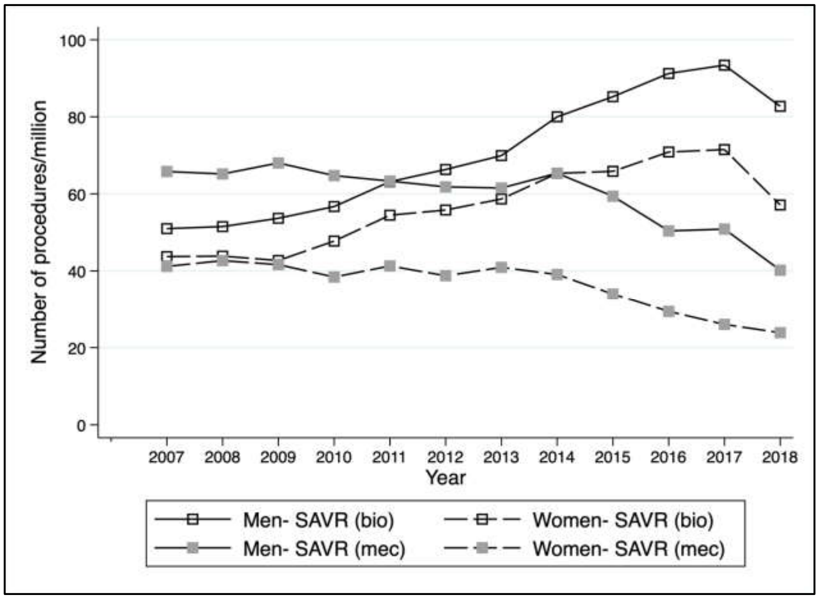

Number of procedures per million of men or women and year in Spain. Men- SAVR: Men who underwent surgical aortic valve replacement. Women- SAVR: Women who underwent surgical aortic valve replacement. Bio: bioprosthesis. Mec: mechanical prosthesis.

Supplemental Figure S4. Changes in SAVR depending on age.

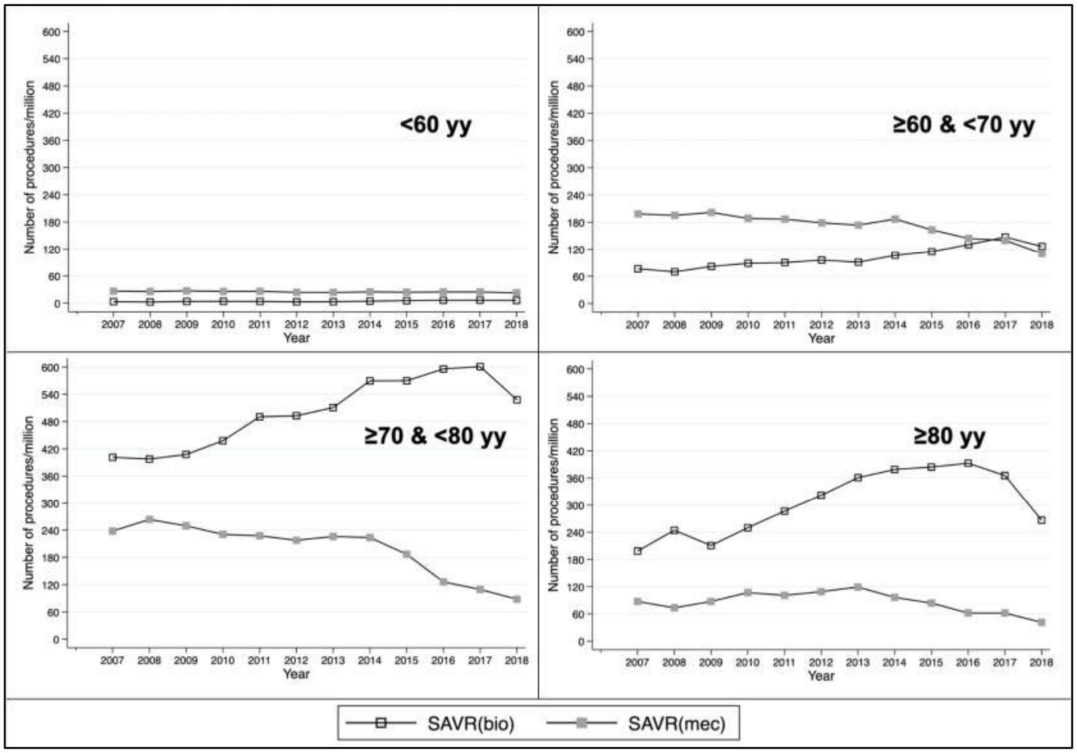

SAVR: Surgical aortic valve replacement. Among patients between 60 and 70, mechanical valves were more commonly implanted than tissue valves except for the las two years. Among patients in their seventies, tissue valves were far more frequent than mechanical valves. Finally, among patients older than 70, tissue valves were more frequent than mechanical.

Supplemental Figure S5. Use of tissue or mechanical valve in the Spanish Autonomous Regions.

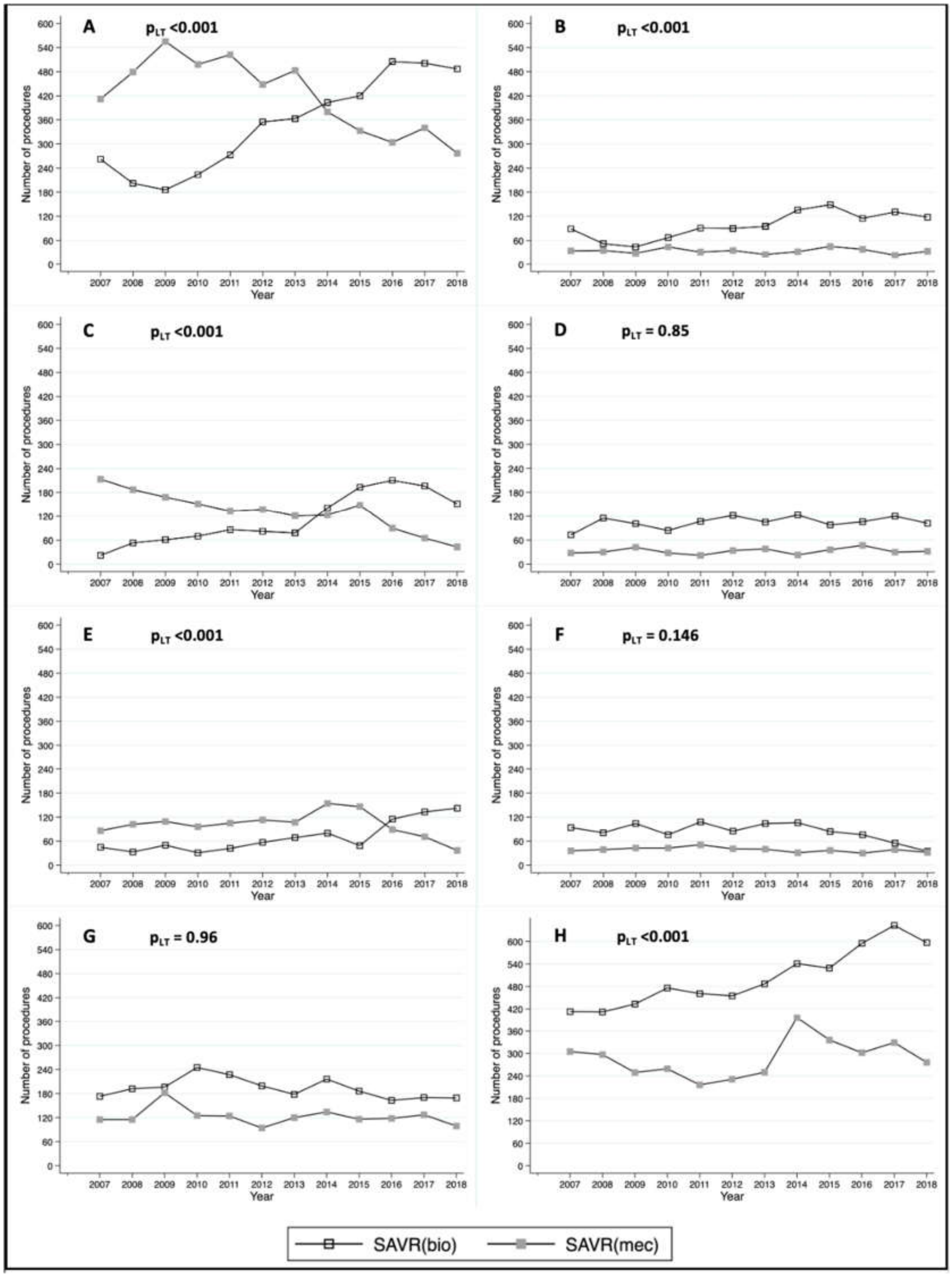

Trends in mechanical or tissue valves in Spain from 2007 to 2018. A) Andalusia B) Aragon C) Principality of Asturias D) Balearic Islands E) Canary Islands F) Cantabria G) Castile & Leon H) Catalonia I) Valencian Community J) Extremadura K) Galicia L) Community of Madrid M) Region of Murcia N) Foral Community of Navarra O) Basque Country. p adjusted for Bonferroni´s correction = 0.003. An increase in tissue valves was detected in all the territories except for Balearic Islands, Cantabria, Castile & Leon and Foral Community of Navarra. Extremadura was the only region in which the use of mechanical valves increased.

Supplemental Figure S5. Use of tissue or mechanical valve in the Spanish Autonomous Regions. Part II.

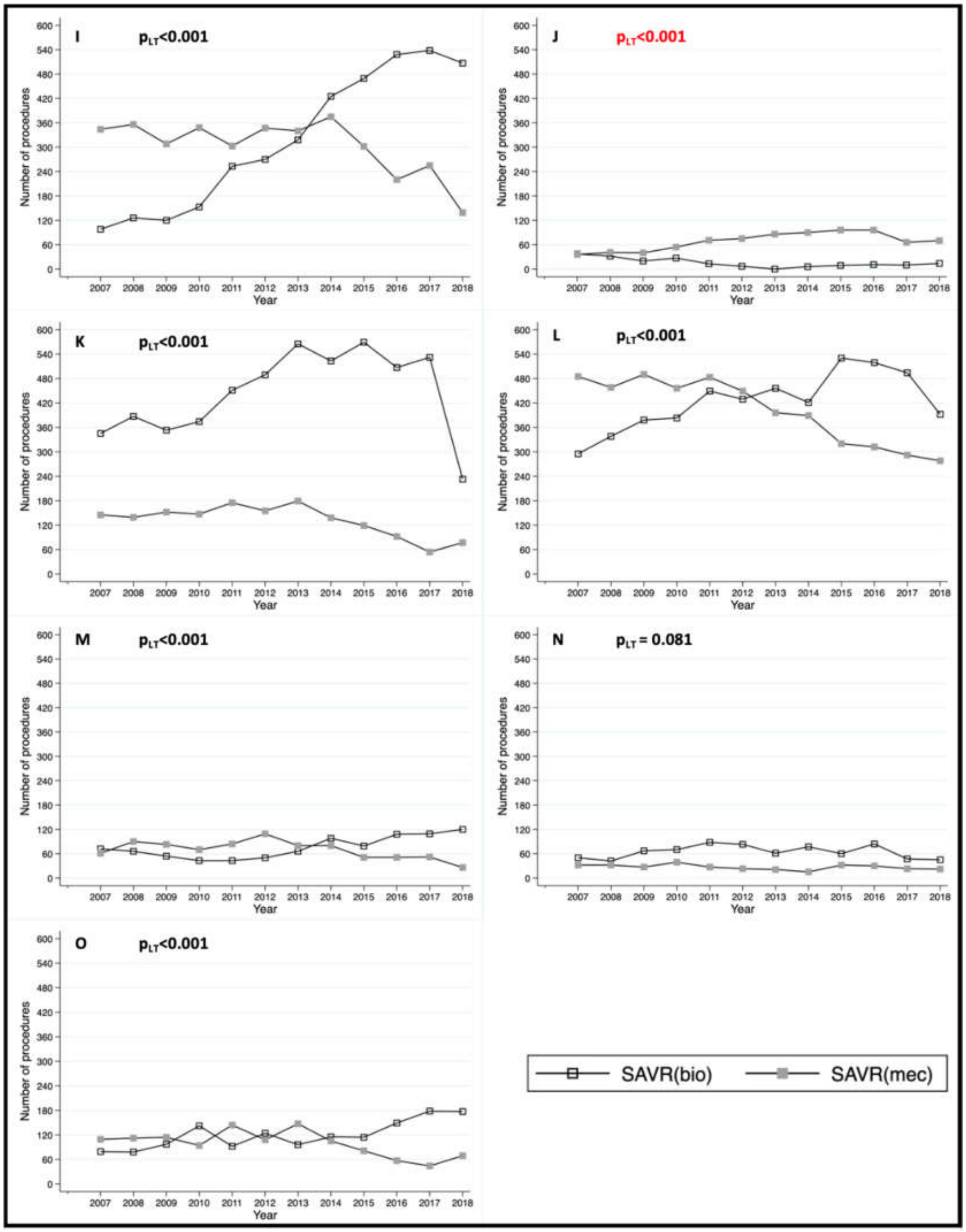

Trends in mechanical or tissue valves in Spain from 2007 to 2018. A) Andalusia B) Aragon C) Principality of Asturias D) Balearic Islands E) Canary Islands F) Cantabria G) Castile & Leon H) Catalonia I) Valencian Community J) Extremadura K) Galicia L) Community of Madrid M) Region of Murcia N) Foral Community of Navarra O) Basque Country. p adjusted for Bonferroni´s correction = 0.003. An increase in tissue valves was detected in all the territories except for Balearic Islands, Cantabria, Castile & Leon and Foral Community of Navarra. Extremadura was the only region in which the use of mechanical valves increased.

Supplemental Figure S6. Predictive model. AUC

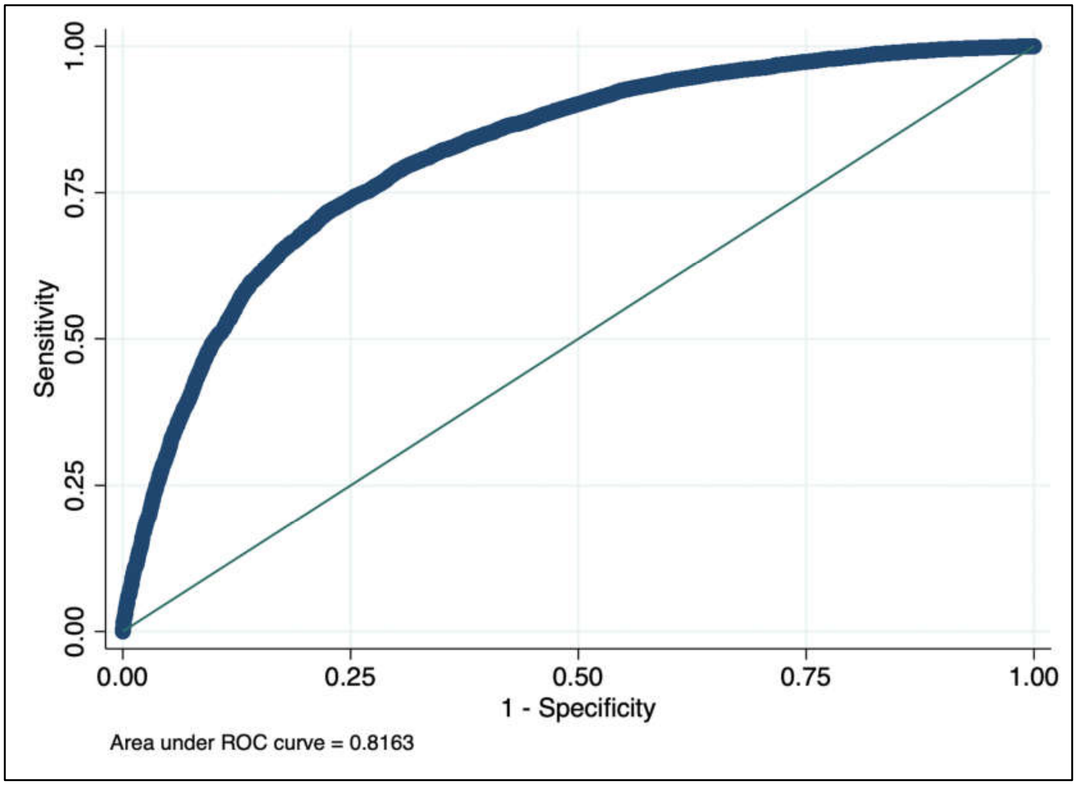

Supplemental Figure S7. Predictive model. Calibration.

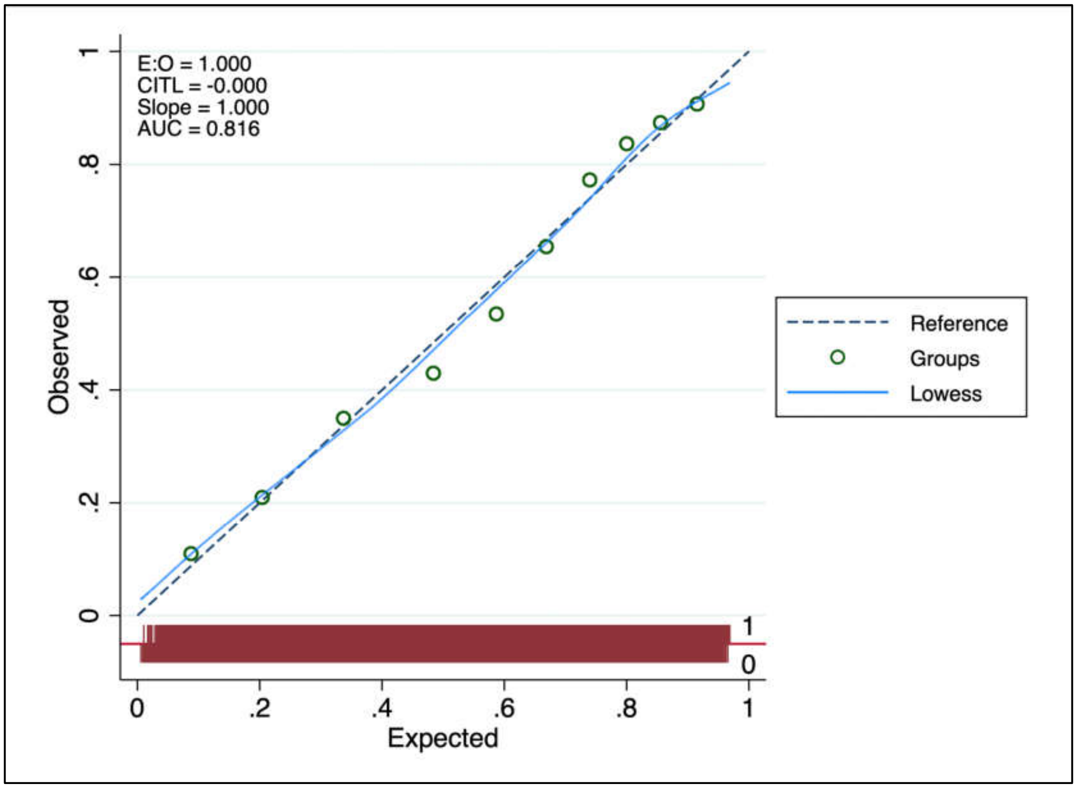

Supplement: Supplementary file 1 [file jcm-10-03209-s001.zip › jcm-1278478-supplementary.pdf]
